# Supplementary material for: Effects of head alignment devices on working memory and postural support during computer work
Source: PLoS One. 2024 Jul 11;19(7):e0306966. doi: 10.1371/journal.pone.0306966 (PMC11239027; doi:10.1371/journal.pone.0306966)
Supplement: S1 Table — (DOCX) [file pone.0306966.s001.docx]

**S1 Table. Repeated measure analysis results of delta wave relative spectral power.**

| Repeated Measure ANOVA | | | | |  | Post Hoc comparisons (Tukey) | | |  |
| --- | --- | --- | --- | --- | --- | --- | --- | --- | --- |
| Dependent Variable | Fixed Factors | Mean ± SD | F | *p* | η_p_² | Variables | | T | *p* |
|  | CPT_U | 63.56 ± 20.32 |  |  |  | CPT_U | CPT_US | -0.86 | 0.671 |
| Fp1 | CPT_US | 65.83 ± 21.43 | 4.34 | 0.017* | 0.108 |  | CPT_T | 1.86 | 0.166 |
|  | CPT_T | 58.93 ± 21.7 |  |  |  | CPT_US | CPT_T | 3.52 | 0.003* |
|  | CPT_U | 59.26 ± 21.91 |  |  |  | CPT_U | CPT_US | -0.87 | 0.662 |
| AF3 | CPT_US | 61.72 ± 24.71 | 5.68 | 0.005* | 0.136 |  | CPT_T | 2.30 | 0.068 |
|  | CPT_T | 52.58 ± 22.91 |  |  |  | CPT_US | CPT_T | 3.41 | 0.005* |
|  | CPT_U | 55.45 ± 22.22 |  |  |  | CPT_U | CPT_US | -0.47 | 0.887 |
| AF4 | CPT_US | 56.96 ± 24.82 | 4.25 | 0.018* | 0.106 |  | CPT_T | 2.30 | 0.069 |
|  | CPT_T | 48.9 ± 21.19 |  |  |  | CPT_US | CPT_T | 2.98 | 0.014* |
|  | CPT_U | 52.75 ± 20.88 |  |  |  | CPT_U | CPT_US | -1.01 | 0.575 |
| AFz | CPT_US | 55.27 ± 22.7 | 3.75 | 0.028* | 0.094 |  | CPT_T | 1.52 | 0.294 |
|  | CPT_T | 48.67 ± 20.84 |  |  |  | CPT_US | CPT_T | 3.17 | 0.009* |
|  | CPT_U | 52.2 ± 18.67 |  |  |  | CPT_U | CPT_US | -0.64 | 0.802 |
| F3 | CPT_US | 53.61 ± 21.29 | 3.18 | 0.048* | 0.081 |  | CPT_T | 1.70 | 0.219 |
|  | CPT_T | 48.56 ± 18.53 |  |  |  | CPT_US | CPT_T | 2.78 | 0.023* |
|  | CPT_U | 51.26 ± 22.1 |  |  |  | CPT_U | CPT_US | -1.77 | 0.193 |
| F4 | CPT_US | 55.97 ± 22.85 | 3.54 | 0.034* | 0.089 |  | CPT_T | 0.57 | 0.835 |
|  | CPT_T | 49.77 ± 20.73 |  |  |  | CPT_US | CPT_T | 3.12 | 0.010* |
|  | CPT_U | 63.32 ± 20.38 |  |  |  | CPT_U | CPT_US | -1.19 | 0.469 |
| F8 | CPT_US | 66.09 ± 20.38 | 5.65 | 0.005* | 0.136 |  | CPT_T | 1.94 | 0.143 |
|  | CPT_T | 58.25 ± 21.60 |  |  |  | CPT_US | CPT_T | 3.71 | 0.002* |
|  | CPT_U | 54.66 ± 19.75 |  |  |  | CPT_U | CPT_US | -1.93 | 0.145 |
| FC5 | CPT_US | 59.15 ± 21.53 | 4.42 | 0.015* | 0.109 |  | CPT_T | 0.97 | 0.603 |
|  | CPT_T | 52.51 ± 19.18 |  |  |  | CPT_US | CPT_T | 2.92 | 0.016* |
|  | CPT_U | 59.02 ± 19.17 |  |  |  | CPT_U | CPT_US | -0.92 | 0.633 |
| Cz | CPT_US | 60.77 ± 21.25 | 3.15 | 0.049* | 0.08 |  | CPT_T | 1.53 | 0.290 |
|  | CPT_T | 56.30 ± 19.84 |  |  |  | CPT_US | CPT_T | 2.65 | 0.032* |
|  | CPT_U | 52.84 ± 17.68 |  |  |  | CPT_U | CPT_US | -1.64 | 0.243 |
| P8 | CPT_US | 56.39 ± 21.56 | 4.34 | 0.017* | 0.108 |  | CPT_T | 1.24 | 0.440 |
|  | CPT_T | 50.05 ± 19.31 |  |  |  | CPT_US | CPT_T | 3.10 | 0.010* |
|  | CPT_U | 58.80 ± 17.55 |  |  |  | CPT_U | CPT_US | -0.42 | 0.909 |
| P3 | CPT_US | 59.59 ± 20.50 | 3.45 | 0.037* | 0.087 |  | CPT_T | 2.06 | 0.113 |
|  | CPT_T | 55.04 ± 18.61 |  |  |  | CPT_US | CPT_T | 2.50 | 0.044* |
|  | CPT_U | 57.42 ± 16.57 |  |  |  | CPT_U | CPT_US | -0.74 | 0.745 |
| P4 | CPT_US | 58.88 ± 20.19 | 3.22 | 0.046* | 0.082 |  | CPT_T | 1.64 | 0.242 |
|  | CPT_T | 54.07 ± 19.51 |  |  |  | CPT_US | CPT_T | 2.69 | 0.028* |

Abbreviations: Fp, prefrontal; AF, anterior frontal; AFz, midline of anterior frontal; F, frontal; FC, frontocentral; Cz, midline of central; P, parietal; CPT, computer; CPT_U, upright CPT workstation; CPT_US, upright support CPT workstation; CPT_T, traction CPT workstation; η_p_², partial eta-squared; SD, standard deviation. * Statistically significant difference: *p*<0.05
