# Supplementary material for: Effects of head alignment devices on working memory and postural support during computer work
Source: PLoS One. 2024 Jul 11;19(7):e0306966. doi: 10.1371/journal.pone.0306966 (PMC11239027; doi:10.1371/journal.pone.0306966)
Supplement: S2 Table — (DOCX) [file pone.0306966.s002.docx]

**S2 Table. Repeated measure analysis results of the alpha wave relative spectral power.**

| Repeated Measure ANOVA | | | | |  | Post Hoc comparisons (Tukey) | | |  |
| --- | --- | --- | --- | --- | --- | --- | --- | --- | --- |
| Dependent Variable | Fixed Factors | Mean ± SD | F | *p* | η_p_² | Variables | | T | *p* |
|  | CPT_U | 8.57 ± 6.24 |  |  |  | CPT_U | CPT_US | -1.40 | 0.353 |
| Fp1 | CPT_US | 9.54 ± 7.52 | 7.17 | 0.001* | 0.166 |  | CPT_T | -3.43 | 0.004* |
|  | CPT_T | 11.44 ± 8.27 |  |  |  | CPT_US | CPT_T | -2.45 | 0.049* |
|  | CPT_U | 9.67 ± 7.48 |  |  |  | CPT_U | CPT_US | -1.35 | 0.376 |
| AF3 | CPT_US | 10.66 ± 8.66 | 6.27 | 0.003* | 0.148 |  | CPT_T | -2.92 | 0.016* |
|  | CPT_T | 12.73 ± 9.28 |  |  |  | CPT_US | CPT_T | -2.49 | 0.046* |
|  | CPT_U | 9.96 ± 6.89 |  |  |  | CPT_U | CPT_US | -0.65 | 0.792 |
| T8 | CPT_US | 10.48 ± 7.84 | 5.27 | 0.007* | 0.128 |  | CPT_T | -2.87 | 0.018* |
|  | CPT_T | 12.45 ± 7.31 |  |  |  | CPT_US | CPT_T | -2.61 | 0.034* |
|  | CPT_U | 18.09 ± 11.53 |  |  |  | CPT_U | CPT_US | -0.19 | 0.980 |
| P8 | CPT_US | 18.31 ± 14.27 | 4.02 | 0.022* | 0.100 |  | CPT_T | -2.12 | 0.101 |
|  | CPT_T | 21.07 ± 15.13 |  |  |  | CPT_US | CPT_T | -2.99 | 0.014* |

Abbreviations: Fp, prefrontal; AF, anterior frontal; T, temporal; P, parietal; CPT, computer; CPT_U, upright CPT workstation; CPT_US, upright support CPT workstation; CPT_T, traction CPT workstation; η_p_², partial eta-squared; SD, standard deviation. * Statistically significant difference: *p*<0.05
