# Supplementary material for: Effects of head alignment devices on working memory and postural support during computer work
Source: PLoS One. 2024 Jul 11;19(7):e0306966. doi: 10.1371/journal.pone.0306966 (PMC11239027; doi:10.1371/journal.pone.0306966)
Supplement: S3 Table — (DOCX) [file pone.0306966.s003.docx]

**S3 Table. Repeated measure analysis results of beta 2 waves relative spectral power.**

| Repeated Measure ANOVA | | | | |  | Post Hoc comparisons (Tukey) | | |  |
| --- | --- | --- | --- | --- | --- | --- | --- | --- | --- |
| Dependent Variable | Fixed Factors | Mean ± SD | F | *p* | η_p_² | Variables | | T | *p* |
|  | CPT_U | 3.45 ± 2.40 |  |  |  | CPT_U | CPT_US | 2.06 | 0.113 |
| Fp1 | CPT_US | 2.77 ± 1.80 | 4.30 | 0.017* | 0.107 |  | CPT_T | 0.03 | 0.999 |
|  | CPT_T | 3.44 ± 1.87 |  |  |  | CPT_US | CPT_T | -2.75 | 0.025* |
|  | CPT_U | 4.81 ± 3.55 |  |  |  | CPT_U | CPT_US | 2.59 | 0.036* |
| AF3 | CPT_US | 3.59 ± 2.80 | 4.29 | 0.017* | 0.107 |  | CPT_T | 1.13 | 0.503 |
|  | CPT_T | 4.42 ± 2.71 |  |  |  | CPT_US | CPT_T | -1.84 | 0.170 |
|  | CPT_U | 5.11 ± 3.87 |  |  |  | CPT_U | CPT_US | 1.56 | 0.274 |
| AF4 | CPT_US | 4.16 ± 2.87 | 3.73 | 0.029* | 0.094 |  | CPT_T | -0.81 | 0.700 |
|  | CPT_T | 5.52 ± 3.34 |  |  |  | CPT_US | CPT_T | -3.41 | 0.004* |
|  | CPT_U | 4.93 ± 2.37 |  |  |  | CPT_U | CPT_US | 2.57 | 0.038* |
| F3 | CPT_US | 4.26 ± 2.38 | 3.62 | 0.032* | 0.091 |  | CPT_T | 0.64 | 0.799 |
|  | CPT_T | 4.78 ± 2.18 |  |  |  | CPT_US | CPT_T | -1.84 | 0.172 |
|  | CPT_U | 5.18 ± 3.16 |  |  |  | CPT_U | CPT_US | 2.66 | 0.031* |
| F4 | CPT_US | 4.02 ± 2.32 | 4.88 | 0.01* | 0.119 |  | CPT_T | 1.14 | 0.499 |
|  | CPT_T | 4.73 ± 2.25 |  |  |  | CPT_US | CPT_T | -2.64 | 0.032* |
|  | CPT_U | 3.67 ± 2.40 |  |  |  | CPT_U | CPT_US | 2.85 | 0.019* |
| F7 | CPT_US | 2.90± 2.14 | 4.26 | 0.018* | 0.106 |  | CPT_T | 2.00 | 0.126 |
|  | CPT_T | 3.08 ± 1.90 |  |  |  | CPT_US | CPT_T | -0.71 | 0.761 |
|  | CPT_U | 3.70± 2.53 |  |  |  | CPT_U | CPT_US | 2.28 | 0.072 |
| F8 | CPT_US | 2.94 ± 1.94 | 4.82 | 0.011* | 0.118 |  | CPT_T | -0.51 | 0.869 |
|  | CPT_T | 3.88 ± 2.28 |  |  |  | CPT_US | CPT_T | -3.39 | 0.005* |
|  | CPT_U | 4.95 ± 2.59 |  |  |  | CPT_U | CPT_US | 3.50 | 0.004* |
| FC5 | CPT_US | 4.08 ± 2.57 | 6.04 | 0.004* | 0.144 |  | CPT_T | 0.78 | 0.718 |
|  | CPT_T | 4.76 ± 2.23 |  |  |  | CPT_US | CPT_T | -2.31 | 0.067 |
|  | CPT_U | 4.60 2.55 |  |  |  | CPT_U | CPT_US | 2.45 | 0.049* |
| P8 | CPT_US | 3.83 ± 2.61 | 4.29 | 0.017* | 0.107 |  | CPT_T | 1.49 | 0.309 |
|  | CPT_T | 4.30± 2.33 |  |  |  | CPT_US | CPT_T | -1.76 | 0.197 |
|  | CPT_U | 4.78 ± 2.66 |  |  |  | CPT_U | CPT_US | 2.53 | 0.042* |
| C3 | CPT_US | 4.21 ± 2.91 | 3.66 | 0.031* | 0.092 |  | CPT_T | 1.34 | 0.384 |
|  | CPT_T | 4.52 ± 2.72 |  |  |  | CPT_US | CPT_T | -1.47 | 0.320 |

Abbreviations: Fp, prefrontal; AF, anterior frontal; F, frontal; FC, frontocentral; P, parietal; C, central; CPT, computer; CPT_U, upright CPT workstation; CPT_US, upright support CPT workstation; CPT_T, traction CPT workstation; η_p_², partial eta-squared; SD, standard deviation. * Statistically significant difference: *p*<0.05
