# Supplementary material for: Effects of head alignment devices on working memory and postural support during computer work
Source: PLoS One. 2024 Jul 11;19(7):e0306966. doi: 10.1371/journal.pone.0306966 (PMC11239027; doi:10.1371/journal.pone.0306966)
Supplement: Attachment — Submitted filename: 20240222_Response to Reviewers.docx [file pone.0306966.s004.docx]

**Journal requirements:**

1. When submitting your revision, we need you to address these additional requirements.

Please ensure that your manuscript meets PLOS ONE's style requirements, including those for file naming. The PLOS ONE style templates can be found at 
https://journals.plos.org/plosone/s/file?id=wjVg/PLOSOne_formatting_sample_main_body.pdf and 
https://journals.plos.org/plosone/s/file?id=ba62/PLOSOne_formatting_sample_title_authors_affiliations.pdf.

R: The manuscript has been revised to meet the style requirements of PLOS ONE.

2. Thank you for stating the following financial disclosure: 
 [This research was supported by a grant from the National Research Foundation of
Korea (NRF) grant funded by the Korean government (MSIT) (No. 2020R1A2C1004355; No. 2022R1F1A1062766).].  
Please state what role the funders took in the study. If the funders had no role, please state: ""The funders had no role in study design, data collection and analysis, decision to publish, or preparation of the manuscript."" 
If this statement is not correct you must amend it as needed. 
Please include this amended Role of Funder statement in your cover letter; we will change the online submission form on your behalf.

R: The statement “The funders had no role in study design, data collection and analysis, decision to publish, or preparation of the manuscript." was included in the coverletter.

3. In the online submission form, you indicated that [Insert text from online submission form here]. 
All PLOS journals now require all data underlying the findings described in their manuscript to be freely available to other researchers, either 1. In a public repository, 2. Within the manuscript itself, or 3. Uploaded as supplementary information.
This policy applies to all data except where public deposition would breach compliance with the protocol approved by your research ethics board. If your data cannot be made publicly available for ethical or legal reasons (e.g., public availability would compromise patient privacy), please explain your reasons on resubmission and your exemption request will be escalated for approval.

R: We uploaded the data described in the manuscript to a public repository at the following link: https://www.kaggle.com/datasets/jyjung95/physiology-and-cognition

4. PLOS requires an ORCID iD for the corresponding author in Editorial Manager on papers submitted after December 6th, 2016. Please ensure that you have an ORCID iD and that it is validated in Editorial Manager. To do this, go to ‘Update my Information’ (in the upper left-hand corner of the main menu), and click on the Fetch/Validate link next to the ORCID field. This will take you to the ORCID site and allow you to create a new iD or authenticate a pre-existing iD in Editorial Manager. Please see the following video for instructions on linking an ORCID iD to your Editorial Manager account: <https://www.youtube.com/watch?v=_xcclfuvtxQ>.

R: ORCID ID was linked.

5. Please include your full ethics statement in the ‘Methods’ section of your manuscript file. In your statement, please include the full name of the IRB or ethics committee who approved or waived your study, as well as whether or not you obtained informed written or verbal consent. If consent was waived for your study, please include this information in your statement as well.

R: This study was approved by the Gachon University Bioethics Committee (IRB). We obtained written informed consent from all participants. We included the relevant contents in the 'Method' section.

6. We note that Figure 2 and 3 in your submission contain copyrighted images. All PLOS content is published under the Creative Commons Attribution License (CC BY 4.0), which means that the manuscript, images, and Supporting Information files will be freely available online, and any third party is permitted to access, download, copy, distribute, and use these materials in any way, even commercially, with proper attribution. For more information, see our copyright guidelines: http://journals.plos.org/plosone/s/licenses-and-copyright.

[PLOS Copyright Guidelines](http://journals.plos.org/plosone/s/licenses-and-copyright).
We require you to either (1) present written permission from the copyright holder to publish these figures specifically under the CC BY 4.0 license, or (2) remove the figures from your submission:
 a. You may seek permission from the original copyright holder of Figure 2 and 3 to publish the content specifically under the CC BY 4.0 license. 
We recommend that you contact the original copyright holder with the Content Permission Form (http://journals.plos.org/plosone/s/file?id=7c09/content-permission-form.pdf) and the following text:
“I request permission for the open-access journal PLOS ONE to publish XXX under the Creative Commons Attribution License (CCAL) CC BY 4.0 (http://creativecommons.org/licenses/by/4.0/). Please be aware that this license allows unrestricted use and distribution, even commercially, by third parties. Please reply and provide explicit written permission to publish XXX under a CC BY license and complete the attached form.”

Please upload the completed Content Permission Form or other proof of granted permissions as an ""Other"" file with your submission. 
 
In the figure caption of the copyrighted figure, please include the following text: “Reprinted from [ref] under a CC BY license, with permission from [name of publisher], original copyright [original copyright year].”
b. If you are unable to obtain permission from the original copyright holder to publish these figures under the CC BY 4.0 license or if the copyright holder’s requirements are incompatible with the CC BY 4.0 license, please either i) remove the figure or ii) supply a replacement figure that complies with the CC BY 4.0 license. Please check copyright information on all replacement figures and update the figure caption with source information. If applicable, please specify in the figure caption text when a figure is similar but not identical to the original image and is therefore for illustrative purposes only.

R: The figures 2 and 3 are not copyrighted images and are original images obtained through the experiment. We can provide the raw graphic file shown in Figure 2 (revised as figure 3) created directly using an illustrator program. As shown below, Figure 3 (revised as Figure 4) shows brain-wave images generated through the BrainMap program (LAXTHA Inc, Korea).

The brain map image generated through the BrainMap program

The raw graphic image created by illustrator program

Authors’ Responses to Reviewers’ Comments:

Thank you very much for your valuable comments and suggestions. We carefully revised the manuscript following the reviewer’s recommendations accordingly. Point-by-point responses are as follows:

Comments and Suggestions for Authors:

**-----------------------------------------------------------------------------------------------------**

**Reviewer #1:**

1, The study is interesting and meaningful. People spend large amount of time in working on computer.

Just a minor comment: How long is the adaptation period at the workstation?

It is suggested to use a chart or diagram to illustrate the experimental protocol with the timeline.

R1_1: Thank you very much for your valuable comments and suggestions.

The adaptation period was 5 min. A flow chart of experimental protocol with the timeline was included in the Method as shown below.

**Reviewer #2:**

The purpose of the study was to examine the effect of head alignment on the cognitive productivity and physical health of computer workers. The study is interesting but needs clarification. Some comments are below.

1, Title – Please, consider revising the title. It is not linked to the rationale of the manuscript. The term “assistance” seems not to be appropriate to represent the effect of postural orientation on cognitive tasks. It is confusing. The terms “productivity” and “physical healthy” are generic. The title should reflect the main findings/message of the study.

R2_1: The title has been revised based on the reviewer’s recommendation.

“Application of head during computer works to enhance working memory and postural support – EEG study.”

2, Abstract – this section needs revision. Instead of asking a question, it is necessary to clarify the meaning of postural assistance to the purpose of the study. The statement “all workstations had a substantial effect on postural assistance” needs context to understand. In general, the letter p for statistics is always represented in lowercase. Please, clarify what “physical stability” means. Instead of being generic with “brain dynamics”, please, include at least one explanation about the brain activation results based on frequency analyses. What is the main point and message?

R2_2: We have revised the first question-based sentence and inappropriate sentences in the abstract according to the reviewer’s opinion. The modified sentences are highlighted as shown below.

“The most common risk factor of computer workers is poor head and neck posture. Therefore, upright seated posture has been recommended repeatedly. However, maintaining an upright seated posture is challenging during computer work and induces various complaints, such as fatigue and discomfort, which can interfere working performance. Therefore, it is necessary to maintain an upright posture without complaints or intentional efforts during long-term computer work. Alignment devices are an appropriate maneuver to support postural control for maintaining head-neck orientation and reduce head weight. This study aimed to demonstrate the effects of workstations combined with alignment device on head-neck alignment, muscle properties, comfort and working memory ability in computer workers, and to determine the most effective maneuver for productivity and safety at work. Computer workers (n=37) participated in a total of three sessions (upright computer (CPT_U), upright support computer (CPT_US), traction computer (CPT_T) workstations). The craniovertebral angle, muscles tone and stiffness, visual analog discomfort scale score, 2-back working memory performance, and electroencephalogram signals were measured. All three workstations had a substantial effect on maintaining head-neck alignment (p< 0.001), but only CPT_US showed significant improvement on psychological comfort (p=0.04) and working memory performance (p=0.021), which is consistent with an increase in delta power. However, CPT_U showed the increased beta 2 activity, discomfort, and false rates compared to CPT_US. CPT_T showed increased alpha and beta 2 activity and decreased delta activity, which are not conductive to working memory performance. In conclusion, CPT_US can effectively induce efficient neural oscillations without causing any discomfort by increasing delta and decreasing beta 2 activity for working memory tasks.”

3, Introduction – this section addresses the usability of workstations for computer workers focusing on musculoskeletal impairments and reduced cognitive performance. Line 65 – Please, clarify what components of physical health and cognitive performance could be impaired by postural orientations (e.g., upright sitting). How postural stabilization can influence cognitive performance?

R2_3: The section introduces the psychological issues that may arise from an excessive focus on upright sitting. Slater et al. reported that people who were asked to work hard to achieve correct posture (sit up straight) to prevent pain had more anxiety by avoiding incorrect posture (such as swayback), suggesting that helping individuals adopt a more relaxed and comfortable posture may be more beneficial for symptom relief than constantly striving for upright sitting and the excessive effort to maintain upright posture may lead to further worry and increased discomfort from unaccustomed posture (Slater et al., 2019). Therefore, our study focused on the cognitive performance that was affected by not postural alignment (upright sitting) but the effort required to maintain an upright posture, which can impact cognitive performance.

- Slater, D., Korakakis, V., O’Sullivan, P., Nolan, D., O’Sullivan, K., 2019. “Sit Up Straight”: Time to Re-evaluate. J Orthop Sports Phys Ther 49, 562–564. https://doi.org/10.2519/jospt.2019.0610

4, Introduction - Line 75 – It is necessary to define the concepts of physical health and cognitive productivity, as their meanings are unclear at present.

R2_4: In this study, the physical health is the effects of maintaining head-neck alignment and preventing forward head posture which is anticipated with the workstation combined with cervical traction. And cognitive productivity refers to the complex cognitive functions of working memory ability required for arousal, attention, and memory performance. We have also added the definitions of "physical health" and "cognitive productivity" to the text for more clarity.

5, Introduction - Lines 77-84 – The introduction did not provide sufficient background to understand the relationship between postural stability, alignment devices (cervical traction), and cognitive performance.

R2_5: We have stated more specifically the relationship between upright sitting, cervical traction, and cognitive performance in the Introduction section as the reviewer recommended. The cognitive effects of upright sitting are explained through embodiment cognition which of cognition and emotion proposes a bidirectional relationship between the physical body and psychological states (Niedenthal, 2007).

According to the recent research, upright posture is associated with high-frequency brain activity (beta and gamma waves) compared to the slouched posture, which is linked to increased alertness. Moreover, upright posture demonstrated its cognitive effects by increased feelings of pride and confidence in thoughts (Briñol et al., 2009; Carney et al., 2015; Stepper and Strack, 1993).

The cervical traction helps to maintain postural alignment and stability in an upright but also affects brain activity by modulating the autonomic nervous system. It has also been reported that a decreased heart rate occurs due to the activation of the vagal nerve and inhibition of sympathetic nerves during cervical traction (which may be related with delta power increase and alpha power decrease) (Pan et al., 2012). These physiological changes, including increased delta waves and decreased alpha waves, can affect perception, attention, and brain activity (Maraver et al., 2020; Ricci et al., 2020; Schachter, 2004; Sharon et al., 2021).

- Niedenthal, P.M., 2007. Embodying Emotion. Science 316, 1002–1005. https://doi.org/10.1126/science.1136930
- Briñol, P., Petty, R.E., Wagner, B., 2009. Body posture effects on self-evaluation: A self-validation approach. European Journal of Social Psychology 39, 1053–1064. https://doi.org/10.1002/ejsp.607
- Carney, D.R., Cuddy, A.J.C., Yap, A.J., 2015. Review and summary of research on the embodied effects of expansive (vs. Contractive) nonverbal displays. Psychological Science 26, 657–663. https://doi.org/10.1177/0956797614566855
- Stepper, S., Strack, F., 1993. Proprioceptive determinants of emotional and nonemotional feelings. Journal of Personality and Social Psychology 64, 211–220. https://doi.org/10.1037/0022-3514.64.2.211
- Pan, P.J., Tsai, P.H., Tsai, C.C., Chou, C.L., Lo, M.T., Chiu, J.H., 2012. Clinical response and autonomic modulation as seen in heart rate variability in mechanical intermittent cervical traction: a pilot study. J Rehabil Med 44, 229–234. https://doi.org/10.2340/16501977-0927
- Maraver, M.J., Steenbergen, L., Hossein, R., Actis-Grosso, R., Ricciardelli, P., Hommel, B., Colzato, L.S., 2020. Transcutaneous vagus nerve stimulation modulates attentional resource deployment towards social cues. Neuropsychologia 143, 107465. https://doi.org/10.1016/j.neuropsychologia.2020.107465
- Ricci, L., Croce, P., Lanzone, J., Boscarino, M., Zappasodi, F., Tombini, M., Di Lazzaro, V., Assenza, G., 2020. Transcutaneous Vagus Nerve Stimulation Modulates EEG Microstates and Delta Activity in Healthy Subjects. Brain Sciences 10, 668. https://doi.org/10.3390/brainsci10100668
- Schachter, S.C., 2004. Vagus nerve stimulation: mood and cognitive effects. Epilepsy & Behavior, The Neuropsychology of Epilepsy 5, 56–59. https://doi.org/10.1016/j.yebeh.2003.11.007
- Sharon, O., Fahoum, F., Nir, Y., 2021. Transcutaneous Vagus Nerve Stimulation in Humans Induces Pupil Dilation and Attenuates Alpha Oscillations. J. Neurosci. 41, 320–330. https://doi.org/10.1523/JNEUROSCI.1361-20.2020

6, Introduction - Line 80 – Please, be more specific in the definition of “physical and cognitive aspects” and “effective strength for productivity and safety at work” (line 81).

R2_6: We have revised "physical and cognitive aspects" to "physical (head-neck alignment and muscle properties) and cognitive (comfort and working memory ability) aspects" for clarity. Additionally, "effective strength for productivity" has been specified as "effective cervical traction strength for working memory performance" for better understanding.

7, As said, the title, abstract, and introduction sections seem not to be linked. The purpose of the study presented in the abstract seems different than the introduction section. Please, provide the hypotheses for all variables mentioned in the last paragraph of this section (lines 81-84).

R2_7: To ensure consistency of the Abstract and the Introduction as the reviewer commented, we revised the sentence to “the purpose of this study was to demonstrate the effects of workstations combined with traction device on head-neck alignment, muscle properties, comfort and working memory ability in computer workers.”.

The hypothesis of this study has been added in the manuscript as follows:

1. For computer workers, workstations equipped with a traction device are expected to affect head-neck alignment, muscle tone and stiffness, working memory capacity, and comfort.

2. The strength of support from a traction device can have varying effects on head-neck alignment, muscle tone and stiffness, working memory ability, and comfort.

8, Materials and Methods – Lines 87-88 – the first sentence of this section is odd.

R2_8: There was a grammatical error in that sentence, and it was corrected as follows.

This study used a crossover repeated measures design to evaluate the effects of the workstations on cognitive and physical health.

9, Materials and Methods – Lines 88-90 – Please, provide the reference for sample size calculation.

R2_9: The sample size for this study was determined based on the previous study (Jung et al., 2020)

that investigated changes in working memory ability according to posture changes. The sample size for this study was 34 for α error probability of 0.05 and statistical power of 0.95 using G-power. Considering a potential dropout rate of 10%, 3 more participants were recruited.

- Jung, J.-Y., Cho, H.-Y., Kang, C.-K., 2020. Brain activity during a working memory task in different postures: an EEG study. Ergonomics 63, 1359–1370. https://doi.org/10.1080/00140139.2020.1784467

10, Materials and Methods – Line 106 – Please, provide the minimum information to understand the experimental setup without being conducted to another study. Please, provide a figure or picture of the experimental procedures. What were the instructions provided to the participants?

R2_10: As shown in the following figure, a flow chart of the experimental protocol was added based on a reviewer's suggestion.

After the experimental settings such as chair and monitor height were completed, participants were asked to sit with both hands and elbows positioned on the desk. They were instructed to maintain an upright posture and gaze at the center of the monitor without tilting their heads or keeping their backs on the backrest. During the CPT_U task, the traction belt was positioned under the participants' chin without contacting it, and they were instructed to maintain an upright posture. For the CPT_US task, the traction force was set to the weight of the participant's head, and they were asked to maintain an upright posture while placing their head against the traction belt and relax their neck muscles. Similarly, during the CPT_T task, participants were instructed to maintain an upright posture while setting the traction force to 10% of their body weight and relaxing their neck muscles against the traction belt.

During the 2-back task, participants were asked to respond as quickly and accurately as possible, and they were asked not to move during task performance. After completing the task, they were instructed to maintain the same posture even during the post-test assessment.

11, Materials and Methods – Line 111 – Please, specify muscle properties. Were the participants in a sitting position during the familiarization period?

2_11: Muscle properties refer to the tone and stiffness of the muscles. Additionally, the familiarization period (or adaptation period) was conducted while the participants were seated for 5 min.

12, Materials and Methods – Line 118-122 – in the CPT_US condition, does the participant was in constant contact with the traction device? Considering that postural control is influenced by sensorial information and moments of postural instability seem to affect the brain activation, please, provide information on how those systems were affected by each condition. What is being manipulated by these conditions?

R2_12: Participants were wearing a chin strap to equally control sensory input for skin contact for each workstation condition. The traction belt of CPT_US and CPT_T constantly contacted with their chin from the adaptation period to the end of the post-test, while the traction belt of CPT_U was positioned directly under their chin without a direct contact to provide biofeedback for forward bending of the upper body (refer to Figure 3). Therefore, sensory input for the skin contact is equally controlled, with only a difference in supporting strength for each condition.

Furthermore, during the 2-back task, the traction intervention acted constantly with no fluctuations in the weight set on the device (7.3% of body weight and 10% of body weight). As a result, the posture of the subjects remained constantly during task and the movement was monitored in real-time through an external monitor. Therefore, the significant postural instability that could affect brain activity was not observed across all workstations.

13, Materials and Methods – Please, provide information about Figure 1 and Figure 2 e replace when they first appear since they are not related to participants' payment.

R2_13: The information about the figures was provided and the locations of the figures were placed with the relevant sentences in this revised manuscript.

14, Materials and Methods – Task design – Were the numbers displayed on the computer screen? What was the distance between the participants and the screen? "Why was the duration of the stimulus interval so lengthy?" Some studies regarding postural control have shown that stimulus duration and longer intervals may affect postural stability. What instructions were given to the participants?

R2_14: The 2-back task was performed, and the numbers (stimulus) displayed on the center of the computer screen. The distance between the participant and the screen was set at 60 cm horizontally from the eyes as described by Segher et al (2003).
The protocol of this study was followed by previous researches which were the study for demonstrating physiological and clinical response to cervical traction (Pan et al., 2012) and observing changes in subjective mood (alertness, calmness, etc.) and working memory ability in response to postural changes (Jung et al., 2020; Muehlhan et al., 2014). Pan (2012) conducted a protocol involving 10 minutes of cervical traction followed by 5 minutes of rest to examine physiological and clinical differences of cervical traction intervention. Based on this, the workstation was conducted for 10 minutes, with the initial 5 minutes serving as an adaptation period, and the other 5 minutes performing the 2-back task and EEG recording. Additionally, the rest period (stimulus interval) was provided for 5 minutes to eliminate the effects of the workstation. The stimulus duration was based on previous study investigating changes in working memory by postural change (4-minute 2-back task was performed (Muehlhan et al., 2014)). Another research showed 5-minute EEG recording to examine brain waves changes by postural changes (Jung et al., 2020). Therefore, in this study, 2-back task was performed for 5 minutes to acquire stable EEG signals and confirm cognitive effects of the workstation. In addition, previous study has shown significant changes in postural alignment after 5 minutes of computer work (Park et al., 2016). This suggests that maintaining upright posture more than 5 minutes during tasks requires.
Furthermore, the traction device was used to maintain the head position consistently, and participants were instructed not to move except for the finger tapping for 2-back task. Thus, no significant postural instability that could affect cognition was observed. Additionally, all participants were monitored in real-time via an external monitor while performing the 2-back task.

- Seghers, J., Jochem, A., Spaepen, A., 2003. Posture, muscle activity and muscle fatigue in prolonged VDT work at different screen height settings. Ergonomics 46, 714–730. https://doi.org/10.1080/0014013031000090107
- Pan, P.J., Tsai, P.H., Tsai, C.C., Chou, C.L., Lo, M.T., Chiu, J.H., 2012. Clinical response and autonomic modulation as seen in heart rate variability in mechanical intermittent cervical traction: a pilot study. J Rehabil Med 44, 229–234. https://doi.org/10.2340/16501977-0927
- Jung, J.-Y., Cho, H.-Y., Kang, C.-K., 2020. Brain activity during a working memory task in different postures: an EEG study. Ergonomics 63, 1359–1370. https://doi.org/10.1080/00140139.2020.1784467
- Muehlhan, M., Marxen, M., Landsiedel, J., Malberg, H., Zaunseder, S., 2014. The effect of body posture on cognitive performance: a question of sleep quality. Frontiers in Human Neuroscience 8. https://doi.org/10.3389/fnhum.2014.00171
- Park, J., Kang, S., Cynn, H., Jeon, H., 2016. Effect of Tactile Feedback on Trunk Posture and EMG Activity in People With Postural Kyphosis During VDT Work. Physical Therapy Korea 23, 48–56. https://doi.org/10.12674/ptk.2016.23.3.048

15, Materials and Methods – What was the outcome of the cognitive task performance?

R2_15: In this study, participants performed the 2-back working memory task as the cognitive task, which was evaluated by response time and accuracy (false rate). The response time was not significantly changed by the workstations; thus, it was not described in the main text but it was included in Table 5 in the Results.

16, Materials and Methods – How the EEG data acquisition was synchronized with the cognitive task and traction devices.

R2_16: Familiarization with the traction device was carried out before starting the 2-back task. After the adaptation period, the traction was consistently maintained during the 2-back task. EEG recordings began at the beginning of the 2-back task and continued for the 5-min task period. To ensure stable EEG signals, the initial 30 seconds were excluded from the data analysis, and the EEG signals for the subsequent 4 minutes and 30 seconds were analyzed.

17, Materials and Methods – Lines 160-162 – Please, provide the rationale to describe brain activation in terms of frequency bands and the meaning of each band for the study.

R2_17: This study analyzed brain waves in the following bands: delta (0.5–4 Hz), theta (4–8 Hz), alpha (8–13 Hz), beta (13–30 Hz), beta 2 (15-20 Hz), beta 3 (18-40 Hz), and gamma (30–50 Hz). The general characteristics of each band are as follows.

| **Frequency band** | **Brain states** |
| --- | --- |
| Delta | Selectively suppress non-relevant neural activity (Harmony, 2013) |
| Theta | Deeply relaxed, inward focused (Bharti et al., 2021) |
| Alpha | Very relaxed, passive attention (Bharti et al., 2021) |
| Beta | Anxiety dominant, active, external attention (Bharti et al., 2021) |
| Beta 2 | Increases in energy, anxiety (Abhang et al., 2016) |
| Beta 3 | Significant stress, high energy, and high arousal (Abhang et al., 2016) |
| Gamma | Concentration, complex information in consciousness (Başar et al., 2001) |

- Harmony, T., 2013. The functional significance of delta oscillations in cognitive processing. Front. Integr. Neurosci. 7. https://doi.org/10.3389/fnint.2013.00083
- Bharti, M., Bharti, P., Kumar, M., Kumar, P., 2021. Diffuse scattering model for human brain wave (Alpha) propagation prediction during meditation inside a temple, through ray tracing, a hypothetical study. International Multidisciplinary Research Journal 25–29. https://doi.org/10.25081/imrj.2021.v11.7275
- Abhang, P.A., Gawali, B.W., Mehrotra, S.C., 2016. Chapter 3 - Technical Aspects of Brain Rhythms and Speech Parameters, in: Abhang, P.A., Gawali, B.W., Mehrotra, S.C. (Eds.), Introduction to EEG- and Speech-Based Emotion Recognition. Academic Press, pp. 51–79. https://doi.org/10.1016/B978-0-12-804490-2.00003-8
- Başar, E., Başar-Eroglu, C., Karakaş, S., Schürmann, M., 2001. Gamma, alpha, delta, and theta oscillations govern cognitive processes. International journal of psychophysiology 39, 241–248.

18, Materials and Methods – Is VADs a validated test?

R2_18: First of all, we have modified the abbreviation of the Visual Analogue Discomfort Scale from VADS to VAS-D. VAS-D is a modified assessment of discomfort tool derived from the global assessment VAS (Visual Analogue Scale), which comprehensively evaluates discomfort, pain, fatigue, and other factors. It was initially proposed in the study by Harland (2019). The VAS demonstrates very high reliability with an Intra-class Correlation Coefficients (ICC) of 0.97 [95% CI = 0.96 to 0.98] (P E Bijur, 2001). According to Harland (2019), VAS-D shows a strong correlation with VAS (r = 0.54, p < 0.001), and simultaneously reporting a 7.8% higher sensitivity in evaluating discomfort (Harland and Ryan, 2019).

- Bijur, P.E., Silver, W., Gallagher, E.J., 2001. Reliability of the Visual Analog Scale for Measurement of Acute Pain. Academic Emergency Medicine 8, 1153–1157. https://doi.org/10.1111/j.1553-2712.2001.tb01132.x
- Harland, N., Ryan, C., 2019. “It’s Not Pain It’s Discomfort”: Development and Investigation of a Discomfort Measurement Scale. Pain and Rehabilitation - the Journal of Physiotherapy Pain Association 2019, 19–23.

19, Materials and Methods – Lines 173-179 – what were the specific variables for tone and stiffness? Overall, please, provide a complete description of the experimental setup.

R2_19: The tone (Hz) and stiffness (N/m) of the superficial skeletal muscle according to the workstation were measured using a handheld myotonometer with excellent intra and inter-tester reliability (ICC = 0.97) (Myoton AS, Tallinn, Estonia) (Shin et al., 2020). Muscle stiffness is the magnitude of the force against displacement of the fascia tissue and indicates the level of resistance of the myofascial tissue to external forces. Muscle tone is the vibration of the myofascial tissue caused by the resistance of passive stretch.

The skeletal muscles of interest were both side of the suboccipital muscles (SM), levator scapulae (LS), upper trapezius (UT), and sternocleidomastoid (SCM). The exact measurement locations were based on previous studies (Jung et al., 2022). The measurement points as follows: Sub-occipital muscles (SM): SM marked between the middle of the C2 spinous process and the occiput, the point of SM was placed above the atlanto-occipital joint and including rectus capitis posterior (Hamilton et al., 2007; Hong et al., 2020). Upper trapezius (UT): The measuring point of the UT was assessed based on the midpoint of the horizontal line connected from the acromion to the spinous process of the seventh cervical vertebra (Viir et al., 2007). Levator scapulae (LS): LS is marked just above the superior angle of the scapula (Shin et al., 2020). Sternocleidomastoid (SCM): The SCM was examined at a point located midway between the insertion to the anterior surface of manubrium sterni and the mastoid process of the temporal bone (Kocur et al.,2019).

- Shin, H.-J., Kim, S.-H., Hahm, S.-C., Cho, H.-Y., 2020. Thermotherapy Plus Neck Stabilization Exercise for Chronic Nonspecific Neck Pain in Elderly: A Single-Blinded Randomized Controlled Trial. Int J Environ Res Public Health 17, E5572. https://doi.org/10.3390/ijerph17155572
- Jung, J.-Y., Cho, H.-Y., Kang, C.-K., 2022. Effects of a Traction Device for Head Weight Reduction and Neutral Alignment during Sedentary Visual Display Terminal (VDT) Work on Postural Alignment, Muscle Properties, Hemodynamics, Preference, and Working Memory Performance. International Journal of Environmental Research and Public Health 19, 14254. https://doi.org/10.3390/ijerph192114254
- Hamilton, L., Boswell, C., Fryer, G., 2007. The effects of high-velocity, low-amplitude manipulation and muscle energy technique on suboccipital tenderness. International Journal of Osteopathic Medicine 10, 42–49. https://doi.org/10.1016/j.ijosm.2007.08.002
- Hong, J.-H., Lee, D.-H., Kim, S.-E., Seo, D., 2020. Correlation between contraction ratio, endurance, and muscle tone of cervical muscles. Physical Therapy Rehabilitation Science 9, 302–308. https://doi.org/10.14474/ptrs.2020.9.4.302
- Viir, R., Virkus, A., Laiho, K., Rajaleid, K., Selart, A., Mikkelsson, M., 2007. Trapezius muscle tone and viscoelastic properties in sitting and supine positions. SJWEH Supplements 3, 76–80.
- Kocur, P., Wilski, M., Goliwąs, M., Lewandowski, J., Łochyński, D., 2019. Influence of Forward Head Posture on Myotonometric Measurements of Superficial Neck Muscle Tone, Elasticity, and Stiffness in Asymptomatic Individuals With Sedentary Jobs. Journal of Manipulative and Physiological Therapeutics 42, 195–202. https://doi.org/10.1016/j.jmpt.2019.02.005

20, Results – Lines 198-200, 207-209, and others, - Please report the statistics result adequately (F values, p values). For example, in general, the letter p for statistics is always represented in lowercase. Include the effect size values for each analysis.

R2_20: Following the reviewer’s comment, we have revised the statistical results. Additionally, effect sizes were calculated using partial η² and added to the main tables. The supplementary results were also updated.

21, Results – N-back results are unclear. Please, describe how the variables (false rate and response time) were computed in the Methods section. Table 5 – Please, provide the time response of the working memory task in each workstation condition.

R2_21: In the Method section, we have added information for false rate and response time, and we added the response time to Table 5. The false rate was calculated as the proportion of incorrect responses out of 150 stimulus responses (number of false responses/150 x 100). Response time refers to the average time that the participant's response to stimuli appears with the M or N key. The response time are provided as below.

| Repeated Measure ANOVA | | | |  |  |
| --- | --- | --- | --- | --- | --- |
| Dependent Variable | Fixed Factors | Mean ± SD | F | p | Partial η² |
| Response time (ms) | CPT_U | 653.70 ± 189.53 | 0.97 | 0.364 |  |
|  | CPT_US | 677.81 ± 197.39 |  |  | 0.026 |
|  | CPT_T | 663.60 ± 221.12 |  |  |  |

22, Results – Figure 3 – please, provide the meaning of the colors.

R2_22: The color in the brain map image indicates the power spectrum value of each channel. Note that a higher value has the red color. Areas that appear red on the brain map indicate higher brain activity at that frequency.

23, Discussion – A different purpose seems to be stated in the first sentence.

R2_23: As the reviewer commented, the authors rewrote the sentence and added more detail explanation to the Discussion as follows. CPT_U, CPT_US, and CPT_T are designed to assist in maintaining upright posture during computer work while enhancing the cognitive function. To confirm the maintenance of an upright posture, CVA assessment was performed. The higher CVA values (closer to 90°) indicated better upright alignment. As a result, CVA improvement was appeared for all CPT_U, CPT_US, and CPT_T compared to the pre-test (51.98°) after performing 5 minutes of the working memory task. Considering that CVA typically decreases by forward lean during computer work, traction workstations showed significant effects in preventing forward lean and maintaining an upright posture. Therefore, this study found that all three workstations for upright assistance had significant effects on maintaining an upright alignment during working memory task.

24, Discussion – Lines 285-287 – It is necessary to further explore the relationship between an upright posture, CVA, and enhancements in working memory task performance based on the results of the study.

R2_24: To exclude the influence of postural alignment, alignment devices were applied to maintain an upright posture. As a result, all workstations (CPT_U, CPT_US, and CPT_T) contributed effectively to maintain upright posture during cognitive task, and there were no significant differences in postural alignment (CVA) among CPT_U, CPT_US, and CPT_T. However, CPT_U, CPT_US, and CPT_T showed significant influence on participants' psychological mood (comfort).

This comfort feeling has lots of influence on cognitive attention. According to discomfort studies, the uncomfortable experience can lead performance deficits due to leading attention directed away from the task. This distraction may lead to slower responses or slower cognitive processing in task-relevant regions (decrease in cognitive performance) (Sosnowski and Brosnan, 2023; Wine, 1971).
Therefore, comfortable workstation can have a significant impact on working memory performance. In this study, only CPT_US showed significantly improved comfort compared to CPT_U. This suggests that increased support strength in CPT_US compared to CPT_U may influence to comfort. Moreover, the decrease in the false rate of 2-back task in CPT_US compared to CPT_U indicates the close relation between comfort and working memory performance enhancement.

- Sosnowski, M.J., Brosnan, S.F., 2023. Under pressure: the interaction between high-stakes contexts and individual differences in decision-making in humans and non-human species. Anim Cogn 26, 1103–1117. <https://doi.org/10.1007/s10071-023-01768-z>
- Wine, J., 1971. Test anxiety and direction of attention. Psychological Bulletin 76, 92–104. https://doi.org/10.1037/h0031332

25, Discussion – Line 310-317 – the implications of the posture alignments and working memory outcomes are poorly discussed. Please, discuss how head position and additional somatosensory information (traction) affect the postural stability and how it influences the performance of the working memory task. Does postural alignment affect working memory performance including false rate and response time due to the use of the same CNS resources?

R2_25: According to previous research, poorly posture (head facing forward) contributes to slower responses in working memory performance (Jung et al., 2022). Thus, we excluded the influence of poorly posture alignment by workstations (CPT_U, CPT_US, and CPT_T) which make participants into an upright alignment and minimize the impact of posture alignment. In this study, there were no significant differences in CVAs, suggesting that the influence of poorly posture was effectively controlled unlike the previous research.

Postural control is the ability to maintain posture and balance against gravity, and it operates through complex mechanisms including sensory, motor and central nervous system. Especially, the continuous neuromuscular recruitment requires to control the posture (Meier et al., 2019; Willigenburg et al., 2013). Thus, constantly maintaining ideal upright posture is difficult when simultaneously performing cognitive task and requires various efforts.

Therefore, this study focused on differences in the effort required for upright posture control, and cervical traction (external assistance) was employed to increase postural stability with lower motor recruitment. The cervical traction was utilized to increase the base of support (BOS) and the traction force was changed to apply different support strength by de-weighting a head. The support strength can contribute to ease neuromuscular recruitment by de-weighting a head and decreasing motor variability of head and trunk. Furthermore, it also influences to alleviate the conscious burden to maintain an upright posture, which may be induced to concentrate more on cognitive tasks. These effects were confirmed through the decrease in the false rate of the 2-back working memory task in CPT_US compared to CPT_U.

Therefore, both postural alignment and support have an impact on working memory performance. Postural alignment (poor alignment vs. ideal upright alignment) influences response speed, while postural support affects accuracy. This distinction suggests that the postural alignment and support have different relation to cognitive tasks.

- Jung, J.-Y., Cho, H.-Y., Kang, C.-K., 2022. Effects of a Traction Device for Head Weight Reduction and Neutral Alignment during Sedentary Visual Display Terminal (VDT) Work on Postural Alignment, Muscle Properties, Hemodynamics, Preference, and Working Memory Performance. International Journal of Environmental Research and Public Health 19, 14254. https://doi.org/10.3390/ijerph192114254
- Meier, M.L., Vrana, A., Schweinhardt, P., 2019. Low Back Pain: The Potential Contribution of Supraspinal Motor Control and Proprioception. Neuroscientist 25, 583–596. https://doi.org/10.1177/1073858418809074
- Willigenburg, N.W., Kingma, I., Hoozemans, M.J.M., van Dieën, J.H., 2013. Precision control of trunk movement in low back pain patients. Human Movement Science 32, 228–239. https://doi.org/10.1016/j.humov.2012.12.007

26, Discussion – Lines 325-326 – Could you please clarify the evidence that supports this statement? There is no mention of physical health and no hypotheses related to it. Please, revise this concept and bring the hypotheses and discussion about this issue.

R2_26: According to the previous research, human comfort is linked to health and work efficiency. The more comfortable state induced more cognitive performance (as we mentioned in R2_24) (Sosnowski and Brosnan, 2023; Wine, 1971). Therefore, in this study, the comfort of the workstations was evaluated using both subjective and objective methods. The subjective and objective comforts were measured using VAS-D and EEG features (such as delta and alpha), respectively. EEG signals is an effective way of evaluating psychological state. With increased delta activity, this study confirmed that subjective comfort in CPT_US was significantly improved. The existing evidence shows that relaxation is characterized by increased low frequency oscillations. The lower-frequency EEG bands (including delta and theta band) of the frontal pole may indicate more relaxing or comfortable environments and may be associated with positive subjective moods (Norwood et al., 2019). It is also well established that anxiolitic effect of benzodiazepines is accompanied by a decrease in alpha and increase in delta EEG activity (Breimer et al., 1990; Knyazev, 2007). Therefore, we concluded that CPT_US improved comfort level compared to the CPT_U (Table 3), and delta waves can be promoted in physiologically and psychologically relaxed states.

- Sosnowski, M.J., Brosnan, S.F., 2023. Under pressure: the interaction between high-stakes contexts and individual differences in decision-making in humans and non-human species. Anim Cogn 26, 1103–1117. <https://doi.org/10.1007/s10071-023-01768-z>
- Wine, J., 1971. Test anxiety and direction of attention. Psychological Bulletin 76, 92–104. https://doi.org/10.1037/h0031332
- Norwood, M.F., Lakhani, A., Maujean, A., Zeeman, H., Creux, O., Kendall, E., 2019. Brain activity, underlying mood and the environment: A systematic review. Journal of Environmental Psychology 65, 101321. https://doi.org/10.1016/j.jenvp.2019.101321
- Breimer, L.T.M., Hennis, P.J., Burm, A.G.L., Danhof, M., Bovill, J.G., Spierdijk, Joh., Vletter, A.A., 1990. Quantification of the EEG Effect of Midazolam by Aperiodic Analysis in Volunteers. Clin Pharmacokinet 18, 245–253. https://doi.org/10.2165/00003088-199018030-00006
- Knyazev, G.G., 2007. Motivation, emotion, and their inhibitory control mirrored in brain oscillations. Neuroscience & Biobehavioral Reviews 31, 377–395. https://doi.org/10.1016/j.neubiorev.2006.10.004
